# Evaluation of Concentration Changes in Plasma Amino Acids and Their Metabolites in Eventing Horses During Cross-Country Competitions as Potential Performance Predictors

**DOI:** 10.3390/ani15243640

**Published:** 2025-12-17

**Authors:** Flora Philine Reemtsma, Johanna Giers, Stephanie Horstmann, Sabita Diana Stoeckle, Heidrun Gehlen

**Affiliations:** 1 Equine Clinic, Internal Medicine, Freie Universität Berlin, Oertzenweg 19b, 14193 Berlin, Germany; 2 Tierklinik Großmoor, Holzweg 13, 29352 Adelheidsdorf, Germany; 3 German Olympic Committee for Equestrian Sports (DOKR), Freiherr-von-Langen-Straße 15, 48231 Warendorf, Germany

**Keywords:** amino acids, performance diagnostics, equine, exercise, sport horses

## Abstract

Amino acid (AA) metabolism and the adaptation of muscles to increased physical performance are interdependent. This field study examined changes in plasma amino acid (PAA) concentrations and two metabolites (ammonia and urea) before and after cross-country (CC) competitions throughout an entire eventing season and their associations with performance outcomes in eventing horses. Twenty horses from nine different Warmblood breeds were evaluated between two and seven times across 14 international eventing competitions over a 23-week period. A total of 55 rides were sampled. Blood samples were collected at four time points: before the start (TP0), 10 min after completion of the CC ride (TP1), 30 min post-CC (TP2), and the next morning (TP3). A mixed ANOVA revealed significant effects (*p* < 0.05) of calendar week on PAA concentrations for asparagine, ornithine, and proline. Correlations between CC scores and PAA concentrations were observed for 4 of the 25 measured parameters. TP0 leucine concentrations and TP2 histidine concentrations correlated negatively with CC penalty score, whereas TP1 proline concentrations and TP3 alanine concentrations correlated positively with CC penalty score. These results indicate that higher plasma leucine concentrations may contribute to improved performance in eventing horses. Additionally, alanine and proline show potential as performance-related diagnostic biomarkers.

## Simple Summary

Amino acids are essential for muscle metabolism and exercise adaptation in athletic horses. This field study examined changes in plasma amino acid concentrations and two metabolites (ammonia and urea) before and after cross-country (CC) phases during the eventing season, as well as their associations with competition outcomes. Twenty Warmblood horses competed in 14 international events over 23 weeks, resulting in 55 CC rides analyzed with blood samples collected at four time points surrounding each CC test. Concentrations of asparagine, ornithine, and proline varied across the calendar weeks. Higher pre-exercise leucine and post-exercise histidine concentrations were associated with better CC scores, whereas higher post-exercise proline and alanine concentrations correlated with worse performance outcomes. These findings indicate that higher pre-exercise leucine concentrations may reflect a more favorable metabolic profile, although further experimental research is required to determine whether this association is causal. Alanine and proline have shown potential as performance-related diagnostic variables in eventing horses.

## 1. Introduction

The adaptation of skeletal muscles in athletes occurs as a result of physical exercise and training. Muscular plasticity plays a crucial role in regulating functionality, metabolism, and overall performance capacity [1,2]. In horses, repeated exercise stimuli elicit pronounced structural and metabolic remodeling of muscle tissue. These adaptations include an increase in muscle fiber cross-sectional area and capillarization [3], stimulation of aerobic pathways, improved buffering capacity, and augmented mitochondrial volume with prolonged training [4]. Together, these adaptations improve endurance, recovery, and overall performance. Training also influences various systemic and biochemical markers. Hematological parameters, such as blood counts, clotting indices [5,6], and biochemical indicators, are widely used to assess adaptation and health status [7]. Total protein concentrations tend to increase during long-term endurance training [8], whereas proteomic changes occur with intensive conditioning [9]. Similar adaptations have been reported in humans, including enhanced antioxidant capacity in elite athletes [10]. Changes in blood markers related to muscle function and metabolic adaptation in eventing horses during cross-country (CC) competitions have also been documented [11,12]. Furthermore, both the anabolic index and cytokine responses have gained attention as markers of training status in horses, with studies showing training-related shifts in pro- and anti-inflammatory cytokines in endurance conditioning [13], fitness-dependent interleukin-1 receptor antagonist (IL-1Ra) and interleukin-13 (IL-13) responses [14], and discipline-specific changes in the testosterone–cortisol ratio in endurance and racehorses [15].

Amino acid (AA) metabolism is essential for muscular adaptation, as it directly supports protein synthesis, energy production, and cellular signaling—processes necessary for muscle growth, repair, and metabolic flexibility [16]. Exercise-induced alterations in AA metabolism significantly influence skeletal muscle function [17]. Prolonged training decreases long-term methionine, tyrosine, and tryptophan concentrations [18], whereas a 12-week training period in untrained Standardbreds elevates concentrations of branched-chain AAs, phenylalanine, and tyrosine [17]. In contrast, some studies have reported no effect of training on plasma amino acid (PAA) concentration [19]. In humans, both endurance and sprint training induce shifts in PAA profiles over several months [20,21], with implications for endurance capacity [22], fatigue resistance [23], and recovery [10]. In the present study, the term PAA profile refers not only to PAA concentrations but also to the associated nitrogenous metabolites ammonia and urea, thereby capturing a more comprehensive picture of AA turnover during exercise. Our preliminary study in eventing horses demonstrated that PAA concentrations change after CC competitions [24]. Although the acute effects of exercise on AA metabolism are relatively well understood, the specific influence of long-term training adaptation on PAA profiles in horses remains poorly characterized. This knowledge gap is particularly relevant in equestrian disciplines such as eventing, where sustained aerobic capacity, rapid recovery, and muscular endurance are essential for optimal performance.

Therefore, the aim of this study was to identify the PAAs most closely associated with competition results and, consequently, performance outcomes. By monitoring a broad panel of PAAs together with ammonia and urea across an entire competitive season, this study adopts a comprehensive metabolic approach under real competition conditions. It further examines calendar week (CW) dependent changes in PAA concentrations. We hypothesized that the concentrations of selected PAA would change progressively across the competitive season in eventing horses. Our second hypothesis was that a correlation exists between the concentrations of specific PAAs and performance during the CC phase.

## 2. Materials and Methods

This investigation was conducted as part of the “Performance Monitoring Program” of the German Olympic Committee for Equestrian Sports (DOKR), which monitors performance and metabolic condition in German squad eventing horses. Previous findings from this program on cardiovascular health and blood biomarkers have been reported elsewhere [11,12,24,25,26]. Participation in the study was voluntary, non-remunerated, and preceded by written informed consent from both riders and horse owners. The study protocol was approved by the Berlin State Office for Consumer Protection (registration no. 1-02.04.40.2022; VG006).

### 2.1. Horses and Riders

Twenty Sports Horse type horses (10 mares, 10 geldings) aged 7 to 15 years (mean, 11 years), ridden by eight riders and enrolled in the DOKR ‘Performance Monitoring Program,’ participated in this longitudinal observational study. The nine Warmblood breeds represented are listed in Table 1. Horse identification details were retrieved using the FEI database [27]. The horses competed two to eight times during the study period.

### 2.2. Veterinary Examinations

Before enrollment, all horses underwent clinical, electrocardiographic, and echocardiographic assessments performed by a veterinary team to ensure cardiovascular health. Blood samples were collected at the beginning of the season to assess baseline hematological and biochemical values and overall health status. None of the horses had a known history of myopathies or other performance-limiting disorders. Amino acid analyses were not performed prior to the start of the season. At each competition, mandatory pre- and post-competition evaluations were conducted by official FEI veterinarians, confirming that all horses were “fit to compete”. None of the horses were excluded during the study-period for health-related reasons. All horses were free of any medication relevant to doping regulations during the competitions.

### 2.3. Training and Feeding Management

Training regimens were designed by the riders and trainers and typically comprised dressage schooling, jumping, CC sessions, and interval gallops. Each horse was subjected to individualized preparation protocols tailored to its physiological needs and the performance demands of the scheduled competitions. Periods of reduced activity and pasture turnout were also incorporated. The regimens were not standardized or documented in detail.

Feeding practices varied among horses and were tailored to individual workload and condition. The diet consisted of forage and concentrate feeds. No amino acid supplements were administered during the study period.

### 2.4. Competition Conditions and Frequencies

Samples were taken over a 23-week period, between March and September 2022 (CWs 12–35). Participants participated in 14 international eventing competitions held at five venues in Germany and Poland, completing a total of 55 CC starts. Table 2 summarizes the number of rides and competition levels of the rides per CW. The number of competitors per class ranged from 29 to 107, with an average of 51.81 starters. The CC courses had an average length of 3493.91 m, ranging from 2661 to 4455 m. The mean ridden speed was 550 m/min, which varied with competition level. Ground conditions were reported as normal in 47 of the 55 rides (85.5%) and deep in 8 of the 55 rides (14.5%). The course profiles were predominantly flat (30/55 rides, 54.5%), with 7/55 (12.7%) classified as slightly hilly and 18/55 (32.7%) as hilly. Weather conditions during competition were either sunny (25/55 rides, 45.5%) or cloudy (30/55 rides, 54.5%). Temperature on competition days ranged between 13 °C and 29 °C (mean, 20 °C). All competitions were conducted within the temperate continental climate zone of Central Europe.

### 2.5. Performance Outcomes and CC-Penalty Scoring

Of the 55 CC starts, 51 (92.7%) were completed successfully. Two of the four incomplete rides resulted from horse falls at the end of the course, while in the remaining two cases, the rider retired after two refusals. The average CC penalty score was 9.16, ranging from 0 to 44. The mean final placement after all three competition phases was 14.18.

The CC score consisted of the penalty points collected during the ride and was calculated according to the official FEI guidelines. For each refusal, 20 penalty points were added to the rider-horse pair’s account. For every second that the target time was exceeded, 0.4 penalty points were added.

### 2.6. Blood Sampling and Processing

Blood samples were collected by veterinarians in accordance with the protocols and timing previously reported by Giers et al. [11,12] and Reemtsma et al. [24]. Blood was collected at four standardized time points: baseline (TP0, morning before exercise), 10 min post-CC (TP1), 30 min post-CC (TP2), and the following morning between 04:00 and 07:30 (TP3), approximately 24 h (±1 h) after TP0. The interval between TP2 and TP3 ranged from 11 to 21 h, depending on start times. Horses received concentrate feeds between TP0 and TP1, as well as after TP2. Morning samples (TP0 and TP3) were taken prior to concentrate feeding and physical exercise. Horses were granted unrestricted access to forage.

Blood samples were drawn from the jugular vein using Vacutainer systems with 20 G needles and polyethylene terephthalate tubes. Collection sites were disinfected with 1-propanol. For amino acid analysis, EDTA blood was centrifuged within 10 min (1000× *g*, 10 min; EBA 200 portable centrifuge; Andreas Hettich GmbH & Co. KG, Tuttlingen, Germany). Immediately after centrifugation, plasma was transferred into uncoated plastic tubes and stored in the freezer at −20 °C. It was transported in a continuously frozen state to MembraPure GmbH (Hennigsdorf, Germany) within 13 weeks of collection.

### 2.7. Amino Acid Analyses

PAA concentrations were quantified at the MembraPure GmbH laboratory (Henningsdorf, Germany). For each sample, 400 µL EDTA plasma was mixed with 400 µL dilution buffer (containing 100 nmol/mL norleucine as the internal standard) and 200 µL of precipitation solution. Protein precipitation was performed for 30 min at 4 °C, followed by centrifugation at 14,100× *g* for 5 min. AA concentrations were analyzed using an Aracus amino acid analyzer. Analytical sequences began with two calibration standards, followed by ten samples, with an additional standard after every tenth sample and a final calibration at the end of the sequence. AAs and metabolites were detected at a wavelength of 570 nm, except for proline, which was measured at 440 nm.

### 2.8. Data Analysis

Statistical analyses were performed using SPSS (version 29.0.2.0 (20), IBM Corp. Armonk, NY, USA). The Shapiro–Wilk test was used to evaluate whether the data followed a normal distribution. The potential outliers were identified using boxplots. Box’s test for the homogeneity of covariance matrices could not be performed. Sphericity was assessed for each amino acid using Mauchly’s test. When sphericity was violated, the Greenhouse–Geisser epsilon (ε) was considered. If ε exceeded 0.75, the Huynh–Feldt correction was applied; the lower-bound adjustment was used in cases when ε > 0.75 and ε < 0.75. The data were free of outliers. Despite deviations from normality, a mixed ANOVA was applied due to the absence of an appropriate nonparametric alternative. The PAA concentration at each time point was considered the dependent variable. Ride number was modeled as a covariate. Calendar weeks (CW), the number of previous competitions (NC), and competition level (level) were modeled with random intercepts. Individual horse IDs had no significant effect on the outcomes of the mixed ANOVA and were therefore excluded from the model. A separate mixed ANOVA was conducted for each PAA. Statistical significance was defined as *p* < 0.05. Effect sizes are reported as partial eta-squared (η^2^). For orientation in interpreting the effect sizes, the standardized benchmarks proposed by Cohen (small > 0.01, medium > 0.06, and large > 0.14) can be used. Variables with more than 25% missing data were excluded from the analysis because a high proportion of missing values substantially reduced the statistical power of the mixed ANOVA. Bivariate Pearson correlation analyses were performed to assess the linear associations between CC scores and PAA concentrations at individual time points.

### 2.9. Missing Samples

Owing to logistical constraints, including rider-related reasons and scheduling complications, 12 blood samples could not be collected during sample taking at the competition venues (three at TP1, seven at TP2, and two at TP3). Additionally, 22 individual amino acid values were unavailable due to technical limitations. At TP0, the missing values included glutamate (two values), histidine (one value), 1-methylhistidine (one), and ammonia (one). At TP1, values were missing for alanine (two), asparagine (two), glutamate (one), cysteine (two), 1-methylhistidine (one), ornithine (one), and arginine (two). At TP3, one value each for alanine, glutamate, isoleucine, histidine, ornithine, and arginine was unavailable.

## 3. Results

Aspartic acid and α-aminoadipic acid were excluded because the large number of values below the detection limit precluded robust statistical evaluation. Since no TP2 measurements were available for CW 25, this week was excluded from the mixed ANOVA analyses to ensure a complete representation of all planned sampling times. Retaining CW 25 would have led to a systematic imbalance in the data set.

Partial Eta squared (η^2^) values for within-subject effects and interaction effects of independent variables, as assessed with mixed ANOVA, are presented in Table 3. *p*-values for within-subject effects are provided in Table A1 in Appendix A. Mixed ANOVA showed a significant influence of CW on asparagine, ornithine, and proline concentrations. Significant differences in PAA concentrations between CW 35 and other CWs at specific time points were observed for asparagine, proline, ammonia, glycine, and valine. Depending on the number of previous competitions (NC), significant differences in PAA concentrations were observed for valine (TP2), leucine (TP1), proline (TP1 and TP2), and 1-methylhistidine (TP3). Asparagine was the only PAA showing significant differences among competition levels for TP1, with asparagine concentrations in 2* and 3* events being significantly lower than in 4* events. Detailed results of the mixed ANOVA (within-subject effects and parameter estimates) are available in the Supplementary Table S1.

**Figure 1:**
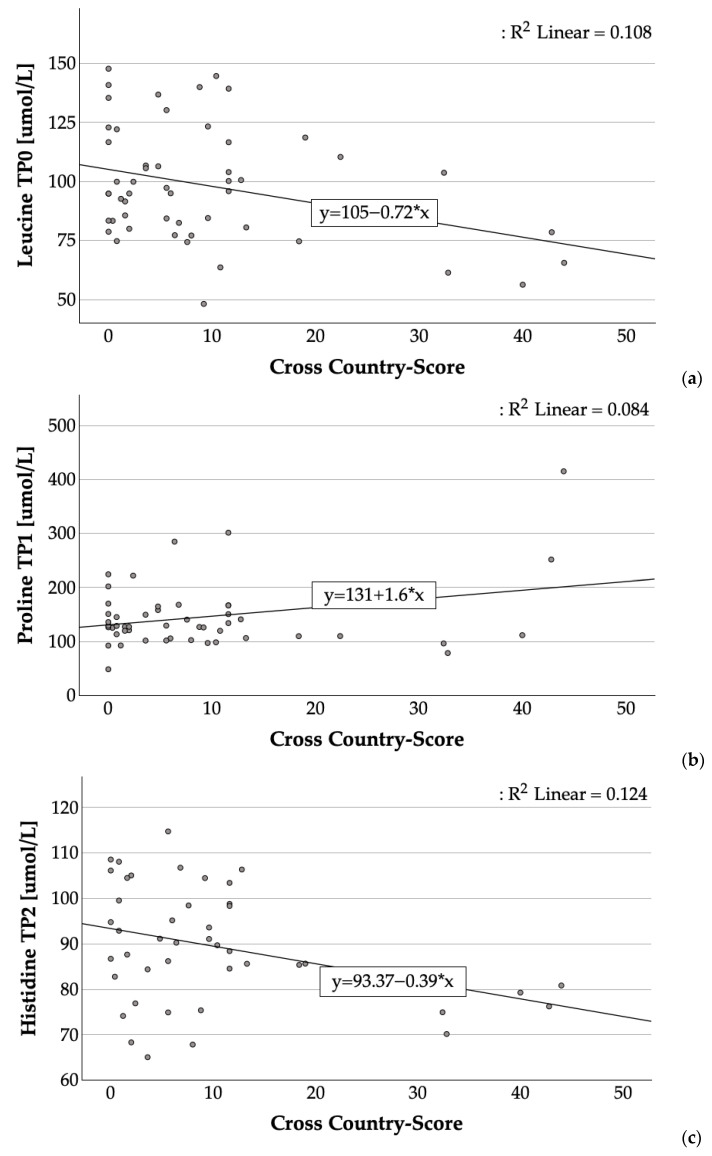

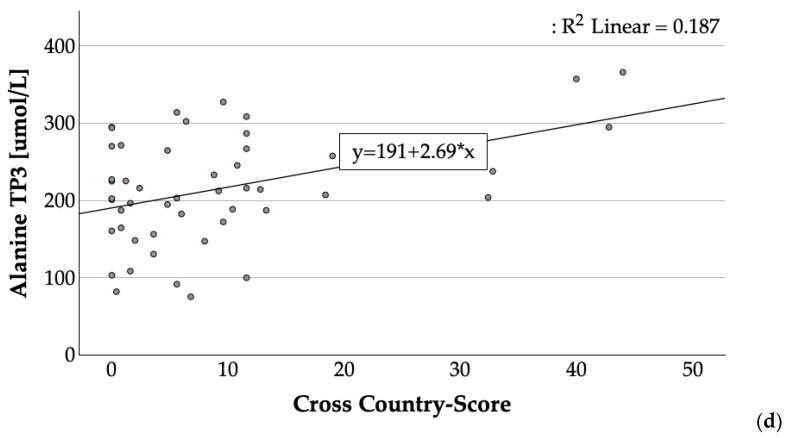
Scatter plots showing the relationship between (**a**) plasma leucine concentrations, (**b**) plasma proline concentrations, (**c**) plasma histidine concentrations, and (**d**) plasma alanine concentrations at specific time points (TP0, TP1, TP2, and TP3) and the CC score for each ride. The regression lines reflect the estimated linear relationship between both variables. The coefficient of determination (R^2^) quantifies the proportion of variability in PAA concentrations accounted for by the CC score.

Correlations between PAA concentrations and CC scores are shown in Table 4. Significant correlations are shown in Figure 1a–d. At TP0, leucine concentrations were negatively correlated with the CC score (r = −0.329, *p* = 0.017). At TP1, proline concentrations were positively correlated (r = 0.29, *p* = 0.043) with the CC score. At TP2, there was a negative correlation between histidine and the CC score (r = −0.353, *p* = 0.018), whereas at TP3, a positive correlation between alanine and the CC score was observed (r = 0.432, *p* = 0.002).

## 4. Discussion

Our first hypothesis, that PAA concentrations change throughout the season, was supported, as this field study on eventing horses revealed significant effects of CW on 3 of the 25 measured parameters. Within-subject effects revealed a significant influence of CW on asparagine concentrations (*p* = 0.031), with pre-exercise asparagine progressively increasing throughout the season. A similar trend was observed in standardbred horses during a 32-week training period [19]. Furthermore, CW had a notable effect on ornithine concentrations. At TP0, ornithine concentrations tended to decrease, whereas at TP2, they increased; however, no significant differences were observed between CW 35 and other weeks. Inconsistent with our results, in standardbred horses undergoing an 8-week training program, the resting plasma ornithine concentration increased over time [28]. A study of young soccer players reported an increase in ornithine concentrations after a 10-month training period [29]. A significant change was observed in proline concentrations between CWs in the within-subject effects (*p* = 0.05). In particular, the TP1 and TP2 concentrations slightly increased during the season. Proline and its metabolite, 4-hydroxyproline, show significant fluctuations in human athletes following intensive training. After running at a high altitude, proline concentrations increased during the recovery phase (30 min post-exercise) and continued to increase 14 days after exercise in human athletes [30], likely reflecting tissue repair and adaptation processes. Consistent with these findings, our previous study demonstrated a post-CC increase in proline concentrations, with the highest concentrations observed 30 min post-exercise (a 155% increase). Proline concentrations remained significantly elevated until the next morning [24]. In contrast to increasing proline concentrations with increasing CWs, a decrease in proline TP1 and TP2 concentrations was observed with an increase in the NC, which supports the finding that training reduces proline concentrations after exercise. Reduced proline plasma concentration in standardbreds due to training has already been investigated, although this change was not statistically significant [19]. Proline has already been identified as a potential biomarker for training adaptation in human athletes because proline serum concentrations decrease during prolonged treadmill exercise [31]. In our initial study, correlations were identified between post-CC lactate and proline concentrations [24].

We accepted our second hypothesis that PAA concentrations correlate with the CC score, as correlations were identified in 4 of the 25 measured parameters. In the current study, proline TP1 concentrations showed a positive correlation with the CC score, indicating that horses with better CC performance exhibited lower post-CC proline concentrations. Assuming that equine athletes with an enhanced level of training are also those in a superior physical condition, proline may serve as a potential biomarker for deriving conclusions regarding horse training conditions. Furthermore, the results of the present study indicate that pre-exercise plasma leucine concentrations were negatively correlated with CC penalty scores, suggesting that horses with higher TP0 leucine concentrations tended to achieve better CC results. Notably, the CC penalty score does not accurately reflect the horses’ physical performance during the CC phase. It serves merely as a reference value that can be influenced by the horse’s fitness and other factors. In addition to the physical condition of the horse, the CC test places technical demands on the rider-horse pair. Thus, the proficiency and experience of both rider and horse in performing such technical tasks must not be underestimated.

Leucine is an important AA involved in physical exertion. Previous studies have provided extensive information regarding leucine fluctuations during physical exercise [24,32,33,34,35,36]; however, no study has yet demonstrated a direct relationship between baseline leucine concentrations and performance outcomes in equine athletes. Leucine is an essential branched-chain AA. It activates the mammalian target of rapamycin complex and adenosine monophosphate-activated protein kinase signaling pathways in equine skeletal satellite cells [37,38]. The combined activation of these pathways leads to increased protein synthesis, mitochondrial function, and the expression of slow myosin heavy chain fibers. These mechanisms are strongly associated with improved endurance capacity [38,39,40,41,42]. Furthermore, leucine enhances oxidative metabolism, resulting in improved aerobic respiration and adenosine triphosphate production [38]. Together, these adaptations improve fatigue resistance, energy efficiency, and recovery. Leucine supplementation also upregulates genes and proteins involved in muscle metabolism and regeneration, thereby supporting muscle adaptation to exercise and potentially reducing injury risk [39,40].

It is acknowledged that sport horses kept in stalls experience management conditions and time-activity budgets that differ substantially from those of horses living in natural environments. These conditions include shorter feeding times, reliance on hay as the primary source of roughage, and reduced exercise duration [43]. Consequently, these factors can influence the metabolic state of sport horses at rest, and they should therefore be considered when interpreting the pre-exercise values in this study. Furthermore, recent research demonstrates that divergent hay feeding systems, together with hay quality, particle size, and the temperament of the horse, can modify feeding rate, meal structure, time spent foraging, and mechanically induced oral disorders [44,45,46,47]. These factors may influence the dynamics of basal nutrient intake and potentially contribute to fluctuations in basal PAA concentrations.

At TP2, a negative correlation was observed between histidine concentrations and the CC penalty score. Alanine concentrations were positively correlated with the CC score at TP3. A previous study reported a positive correlation between alanine and lactate concentrations in eventing horses post-exercise [24]. This indicates that lower post-exercise alanine plasma concentrations are associated with enhanced fitness. Alanine increased by more than 250% post-exercise in the analyzed CC rides [24]. Together, these findings suggest that alanine may serve as an additional biomarker for evaluating training adaptation in horses. The current literature provides limited insight into the relationship between post-exercise alanine concentrations and performance outcomes. However, studies have shown performance-enhancing effects of alanine supplementation. In humans, β-alanine supplementation has been directly linked to improved high-intensity exercise performance [48]. Alanine and histidine interact primarily through their roles in carnosine synthesis, with β-alanine as the rate-limiting precursor and histidine serving as a necessary but non-limiting substrate [49]. Carnosine acts as an important buffer in skeletal muscles, helping to maintain pH during high-intensity exercise, enhance antioxidant activity, and delay fatigue [50,51,52].

Studies on biomarkers have highlighted that endurance conditioning promotes a progressive reduction in pro-inflammatory cytokines [13] and that IL-1Ra and IL-13 exhibit workload- and fitness-dependent modulation across endurance and racehorse populations [14]. Moreover, serum amyloid A has been shown to rise specifically after high-intensity competitive exercise rather than routine training, marking a distinct acute-phase sensitivity [8]. Within this framework, exercise-responsive AAs such as alanine and proline may represent an additional class of metabolic markers that complements cytokine- and acute-phase protein-based indicators of training adaptation. This field study has certain limitations, including the varying number of starts and samples collected per CW, which may have influenced the results. Furthermore, the horses did not follow standardized feeding or training protocols. All horses were examined for cardiovascular health at the beginning of the study; however, they were not for any other metabolic diseases. Baseline PAA values were not measured at the beginning of the season or before the first competition; instead, only other hematologic and biochemical parameters were measured. This study reflects changes within a cohort of already trained, high level eventing horses rather than adaptions occurring during the transition from an untrained to a trained state. All participating horses were in good physical condition, as they had already completed winter training in preparation for the first eventing competition of the year. A control group of untrained horses was not included because sampling was conducted under real competition conditions. Consequently, the first measurement was obtained at the start of the season rather than from completely untrained horses. Potential factors that could influence the results include the lack of a fully standardized time interval between transport and the first sampling, as well as the possible effects of microclimate, stress, or pre-competition routines on PAA concentrations. We identified PAAs, including leucine and histidine, with performance-limiting roles in eventing horses. We also observed a trend in concentration changes for some PAAs over the season; however, these changes could not be attributed to an improvement in training conditions or a state of potential overtraining.

## 5. Conclusions

This study identified higher pre-exercise leucine concentrations as a novel correlate of improved CC performance in trained eventing horses. Moreover, increased post-exercise histidine concentrations were associated with better outcomes, whereas exercise-dependent elevations in alanine and proline were linked to poorer results. These associations require confirmation in independent cohorts. Integrating AA measures with established biomarkers—such as acute-phase proteins, hormonal indices, and anti-inflammatory cytokines—may advance multiparameter tools for monitoring fitness and performance in equine sport.

## Figures and Tables

**Table 1:** Characteristics of the participating horses, including age, breed, sex, and number of competitions participated in during the 2022 season.

Horse	Age	Breed	Sex	Competitions During Season 2022
1	7	Holsteiner	Mare	4
2	7	Oldenburger	Mare	6
3	7	Hanoverian	Mare	6
4	8	Oldenburger	Mare	7
5	8	Westphalian	Gelding	4
6	9	Hanoverian	Gelding	7
7	9	German Sport Horse	Mare	4
8	10	Polish Horse Breeders Association	Mare	7
9	10	Irish Sport Horse	Gelding	4
10	11	Hanoverian	Gelding	5
11	11	Holsteiner	Gelding	6
12	12	Stud Book du Cheval Selle Francais	Mare	6
13	12	Hanoverian	Gelding	6
14	12	Irish Sport Horse	Gelding	6
15	12	Hanoverian	Mare	7
16	14	Hanoverian	Gelding	6
17	14	Holsteiner	Gelding	3
18	15	Hanoverian	Mare	5
19	15	Rheinlander	Mare	2
20	15	Hanoverian	Gelding	8

**Table 2:** Number of rides per calendar week, competed at the different competition levels, including the distribution of starts per FEI test category.

Calendar Week	CCI2 *-S	CCI2 *-L	CCI3 *-S	CCI3 *-L	CCI4 *-S	Total Rides
12	0	0	12	0	0	12
15	1	0	6	0	0	7
16	2	0	0	0	0	2
18	2	1	0	0	7	10
24	0	0	0	0	3	3
25	0	0	2	3	0	5
30	2	0	6	0	0	8
35	1	0	2	0	5	8
Total rides	8	1	28	3	15	55

CCI = Concours Complet International; 2 *, 3 * and 4 * denote the competition level; “-L” = long format; “-S” = short format.

**Table 3:** Partial eta-squared (η^2^) effect sizes of within-subject effects in plasma amino acids and interaction effects of ride number, calendar week (CW), number of previous competitions (NC), and competition level (level). Significant effects (*p* < 0.05) are marked with an asterisk. A–D indicate the statistical method applied for within-subject effects: A, sphericity assumed; B, Greenhouse–Geisser; C, Huyn–Feldt; D, lower bound.

	η^2^ Parameter	η^2^ Ride	η^2^ CW	η^2^ NC	η^2^ Level	Mauchly’s Test
Alanine	0.15 *	0.058	0.125	0.180	0.079	A
Arginine	0.029	0.022	0.069	0.117	0.061	A
Asparagine	0.028	0.029	0.224 *	0.227	0.132	A
Citrulline	0.01	0.013	0.204	0.194	0.098	D
Cysteine	0.059	0.039	0.125	0.199	0.079	D
Glutamine	0.011	0.006	0.133	0.156	0.039	A
Glutamate	0.041	0.034	0.139	0.122	0.017	D
Glycine	0.026	0.03	0.134	0.098	0.027	A
Histidine	0.023	0.016	0.146	0.152	0.057	A
Isoleucine	0.031	0.023	0.124	0.152	0.059	A
Leucine	0.047	0.024	0.102	0.185	0.062	A
Lysine	0.023	0.011	0.085	0.09	0.025	A
Methionine	0.023	0.015	0.149	0.162	0.089	A
1-Methylhistidine	0.003	0.005	0.185	0.402	0.214	D
Ornithine	0.037	0.031	0.233 *	0.111	0.037	A
Phenylalanine	0.045	0.034	0.138	0.118	0.09	A
Proline	0.022	0.008	0.283 *	0.311	0.103	D
Serine	0.032	0.03	0.15	0.095	0.031	A
Taurine	0.042	0.02	0.023	0.299	0.188	D
Threonine	0.040	0.029	0.107	0.131	0.051	A
Tryptophan	0.033	0.026	0.165	0.145	0.062	A
Tyrosine	0.064	0.07	0.164	0.118	0.055	C
Valine	0.033	0.024	0.18	0.247	0.089	A
Ammonia	0.07	0.05	0.208	0.086	0.011	D
Urea	0.032	0.025	0.141	0.145	0.064	A

**Table 4:** Bivariate Pearson correlation coefficients (r) between PAA at TP0, TP1, TP2, and TP3 and the CC score. *p*-values are presented in parentheses. Significant correlations (*p* < 0.05) are shown in bold.

PAA × CC Score	TP0	TP1	TP2	TP3
Alanine	0.227 (0.106)	0.036 (0.808)	−0.079 (0.608)	**0.432 (0.002)**
Arginine	0.015 (0.918)	0.056 (0.709)	0.006 (0.969)	0.141 (0.333)
Asparagine	0.258 (0.065)	0.182 (0.221)	0.108 (0.480)	0.254 (0.075)
Citrulline	−0.147 (.299)	−0.111 (0.450)	−0.223 (0.142)	−0.077 (0.597)
Cysteine	−0.072 (0.612)	−0.124 (0.405)	0.103 (0.503)	−0.089 (0.539)
Glutamine	−0.119 (0.401)	−0.136 (0.351)	−0.209 (0.168)	0.027 (0.852)
Glutamate	−0.026 (0.860)	−0.058 (0.696)	−0.097 (0.525)	0.069 (0.637)
Glycine	−0.231 (0.100)	−0.237 (0.101)	−0.244 (0.106)	−0.175 (0.224)
Histidine	−0.044 (0.761)	−0.090 (0.540)	**−0.353 (0.018)**	0.080 (0.584)
Isoleucine	−0.173 (0.221)	0.086 (0.556)	0.080 (0.599)	0.157 (0.282)
Leucine	**−0.329 (0.017)**	0.038 (0.795)	−0.057 (0.710)	0.109 (0.451)
Lysine	−0.001 (0.994)	−0.038 (0.795)	−0.048 (0.752)	0.221 (0.123)
Methionine	0.033 (0.816)	0.017 (0.910)	0.001 (0.994)	−0.034 (0.813)
1-Methylhistidine	0.109 (0.447)	−0.069 (0.640)	−0.068 (0.658)	0.063 (0.664)
Ornithine	−0.258 (0.065)	−0.106 (0.474)	−0.244 (0.107)	0.104 (0.477)
Phenylalanine	−0.185 (0.190)	−0.133 (0.361)	−0.145 (0.340)	−0.068 (0.640)
Proline	0.010 (0.943)	**0.290 (0.043)**	0.217 (0.151)	0.253 (0.076)
Serine	−0.088 (0.533)	0.001 (0.995)	−0.082 (0.594)	0.035 (0.807)
Taurine	−0.146 (0.301)	−0.134 (0.359)	−0.155 (0.310)	−0.169 (0.240)
Threonine	−0.157 (0.268)	0.066 (0.651)	−0.053 (0.731)	0.031 (0.833)
Tryptophane	−0.154 (0.274)	−0.239 (0.098)	−0.257 (0.089)	−0.118 (0.413)
Tyrosine	−0.189 (0.180)	−0.040 (0.786)	−0.118 (0.442)	−0.102 (0.482)
Valine	−0.147 (0.299)	0.185 (0.203)	0.046 (0.762)	0.167 (0.247)
Ammonia	−0.083 (0.563)	−0.155 (0.288)	−0.105 (0.493)	−0.005 (0.973)
Urea	0.103 (0.467)	0.092 (0.528)	0.053 (0.729)	0.061 (0.674)

## Data Availability

The data presented in this study are available upon request from the corresponding author. These data are not publicly available because of privacy concerns.

## Supplementary Materials

The following supporting information can be downloaded at: https://www.mdpi.com/article/10.3390/ani15243640/s1. Table S1: Within-subject effects and parameter estimates of dependent variables PAA, covariate ride, and independent variables CW, NC, and level.

## Appendix A

**Table A1:** Significances p of within-subject effects in plasma amino acids and interaction effects of ride number, calendar week (CW), number of previous competitions (NC), and competition level (level). Significances (*p* < 0.05) are highlighted in bold. A–D indicate the statistical method applied for within-subject effects: A, sphericity assumed; B, Greenhouse–Geisser; C, Huyn–Feldt; D, lower bound.

	p Parameter	p Ride	p CW	p NC	p Level	Mauchly’s Test
Alanine	** 0.010 **	0.245	0.377	0.644	0.444	A
Arginine	0.588	0.691	0.838	0.955	0.638	A
Asparagine	0.579	0.569	**0.031**	0.350	0.123	A
Citrulline	0.628	0.585	0.148	0.498	0.305	D
Cysteine	0.254	0.353	0.391	0.506	0.406	D
Glutamine	0.930	0.930	0.325	0.791	0.828	A
Glutamate	0.342	0.390	0.340	0.793	0.825	D
Glycine	0.610	0.547	0.317	0.980	0.923	A
Histidine	0.679	0.790	0.279	0.684	0.681	A
Isoleucine	0.534	0.659	0.387	0.645	0.630	A
Leucine	0.345	0.634	0.553	0.611	0.599	A
Lysine	0.648	0.864	0.698	0.988	0.938	A
Methionine	0.653	0.794	0.230	0.755	0.357	A
1-Methylhistidine	0.815	0.733	0.203	0.056	0.071	D
Ornithine	0.452	0.536	**0.024**	0.884	0.844	A
Phenylalanine	0.362	0.491	0.295	0.944	0.350	A
Proline	0.481	0.667	**0.050**	0.159	0.286	D
Serine	0.515	0.545	0.226	0.983	0.895	A
Taurine	0.327	0.499	0.906	0.182	0.091	D
Threonine	0.420	0.570	0.516	0.904	0.711	A
Tryptophan	0.504	0.606	0.161	0.845	0.609	A
Tyrosine	0.206	0.167	0.165	0.943	0.679	C
Valine	0.508	0.634	0.11	0.242	0.360	A
Ammonia	0.210	0.296	0.155	0.904	0.886	D
Urea	0.521	0.627	0.273	0.846	0.581	A
